# Supplementary material for: Dietary Supplementation with Eugenol Nanoemulsion Alleviates the Negative Effects of Experimental Coccidiosis on Broiler Chicken’s Health and Growth Performance
Source: Molecules. 2023 Feb 27;28(5):2200. doi: 10.3390/molecules28052200 (PMC10005078; doi:10.3390/molecules28052200)
Supplement: Supplementary file 1 [file molecules-28-02200-s001.zip › Supplementary Table S1.pdf]

Supplementary Table S1. Composition of basal diet.

Ingredients	Starter (D0–10)	Grower (D11–24)	Finisher (D25–42)
Corn	56	58.97	62.8
Soybean meal	38.5	34.5	30.37
Soybean oil	1.3	2.2	2.9
Dicalcium Phosphate	1.6	1.4	1.3
Calcium carbonate	1.1	1	0.96
Methionine	0.3	0.28	0.25
Lysine	0.25	0.2	0.18
Threonine	0.1	0.6	0.4
Vitamin and mineral premix	0.5	0.5	0.5
Common salt	0.25	0.24	0.22
Sodium bicarbonate	0.1	0.13	0.12
**Calculated composition**			
Metabolizable energy (kcal/kg)	2850	2947	3047
Crude protein (%)	21.8	20.5	18.6
Calcium (%)	0.9	0.82	0.75
Available phosphorus (%)	0.45	0.42	0.39
Digestible methionine (%)	0.66	0.62	0.57
Digestible methionine + cystine (%)	1.02	0.97	0.89
Digestible lysine (%)	1.38	1.26	1.14
