# Supplementary material for: Dietary Supplementation with Eugenol Nanoemulsion Alleviates the Negative Effects of Experimental Coccidiosis on Broiler Chicken’s Health and Growth Performance
Source: Molecules. 2023 Feb 27;28(5):2200. doi: 10.3390/molecules28052200 (PMC10005078; doi:10.3390/molecules28052200)
Supplement: Supplementary file 1 [file molecules-28-02200-s001.zip › Supplementary Table S2.pdf]

Supplementary Table S2. Chemical composition of clove essential oil.

No	Component[a]	Calculated RI [b]	Abundance (%) [c]
1	Limonene	1025	0.2
2	1,8-Cineole	1032	0.1
3	*cis*-Limonene oxide	1136	tr
4	*trans*-Limonene oxide	1147	tr
5	Methyl salicylate	1190	0.1
6	Chavicol	1251	0.2
7	**Eugenol**	1358	**82.2**
8	*β*-Caryophyllene	1415	3.3
9	α-Humulene	1452	1.2
10	**Eugenol acetate**	1520	**12.2**
11	Caryophyllene oxide	1580	0.2
	Total identified (%)		99.7

[a] Compounds are listed in order of their elution from a HP-5MS column.

[b] Linear retention index (RI) on HP-5MS column, experimentally determined using homologous series of C_8-C_{30} alkanes.

[c] Relative percentage values are means of three determinations with a relative standard deviation (RSD%) in all cases below 10%.
